# Genetic assessment in primary hyperoxaluria: why it matters

**DOI:** 10.1007/s00467-022-05613-2

**Published:** 2022-06-13

**Authors:** Giorgia Mandrile, Bodo Beck, Cecile Acquaviva, Gill Rumsby, Lisa Deesker, Sander Garrelfs, Asheeta Gupta, Justine Bacchetta, Jaap Groothoff, on behalf of the OxalEurope Consortium/Erknet Guideline Workgroup On Hyperoxaluria

**Affiliations:** 1 Medical Genetics Unit and Thalassemia Center, San Luigi University Hospital, University of Torino, Orbassano, TO Italy; 2 Institute of Human Genetics, Center for Molecular Medicine Cologne, and Center for Rare and Hereditary Kidney Disease, University Hospital of Cologne, CologneCologne, Germany; 3 Service de Biochimie Et Biologie Moléculaire, Hospices Civils de Lyon, UM Pathologies Héréditaires du Métabolisme Et du Globule Rouge, Lyon, France; 4 Department of Clinical Biochemistry, University College London Hospitals NHS Foundation Trust | UCLH, Kintbury, UK; 5 Department of Pediatric Nephrology, Emma Children’s Hospital, Amsterdam UMC Location University of Amsterdam, Amsterdam, Netherlands; 6 Department of Nephrology, Birmingham Women’s and Children’s NHS Foundation Trust, Birmingham, UK; 7 Reference Center for Rare Renal Diseases, Pediatric Nephrology-Rheumatology-Dermatology Unit, Hospices Civils de Lyon, Femme Mere Enfant Hospital, Lyon 1 University, Bron, France

**Keywords:** Primary hyperoxaluria, Genetics, *AGXT*, *GRHPR*, *HOGA1*

## Abstract

Accurate diagnosis of primary hyperoxaluria (PH) has important therapeutic consequences. Since biochemical assessment can be unreliable, genetic testing is a crucial diagnostic tool for patients with PH to define the disease type. Patients with PH type 1 (PH1) have a worse prognosis than those with other PH types, despite the same extent of oxalate excretion. The relation between genotype and clinical phenotype in PH1 is extremely heterogeneous with respect to age of first symptoms and development of kidney failure. Some mutations are significantly linked to pyridoxine-sensitivity in PH1, such as homozygosity for p.G170R and p.F152I combined with a common polymorphism. Although patients with these mutations display on average better outcomes, they may also present with CKD stage 5 in infancy. In vitro studies suggest pyridoxine-sensitivity for some other mutations, but confirmatory clinical data are lacking (p.G47R, p.G161R, p.I56N/major allele) or scarce (p.I244T). These studies also suggest that other vitamin B6 derivatives than pyridoxine may be more effective and should be a focus for clinical testing. PH patients displaying the same mutation, even within one family, may have completely different clinical outcomes. This discordance may be caused by environmental or genetic factors that are unrelated to the effect of the causative mutation(s). No relation between genotype and clinical or biochemical phenotypes have been found so far in PH types 2 and 3. This manuscript reviews the current knowledge on the genetic background of the three types of primary hyperoxaluria and its impact on clinical management, including prenatal diagnosis.

## Introduction

**Fig. 1:**
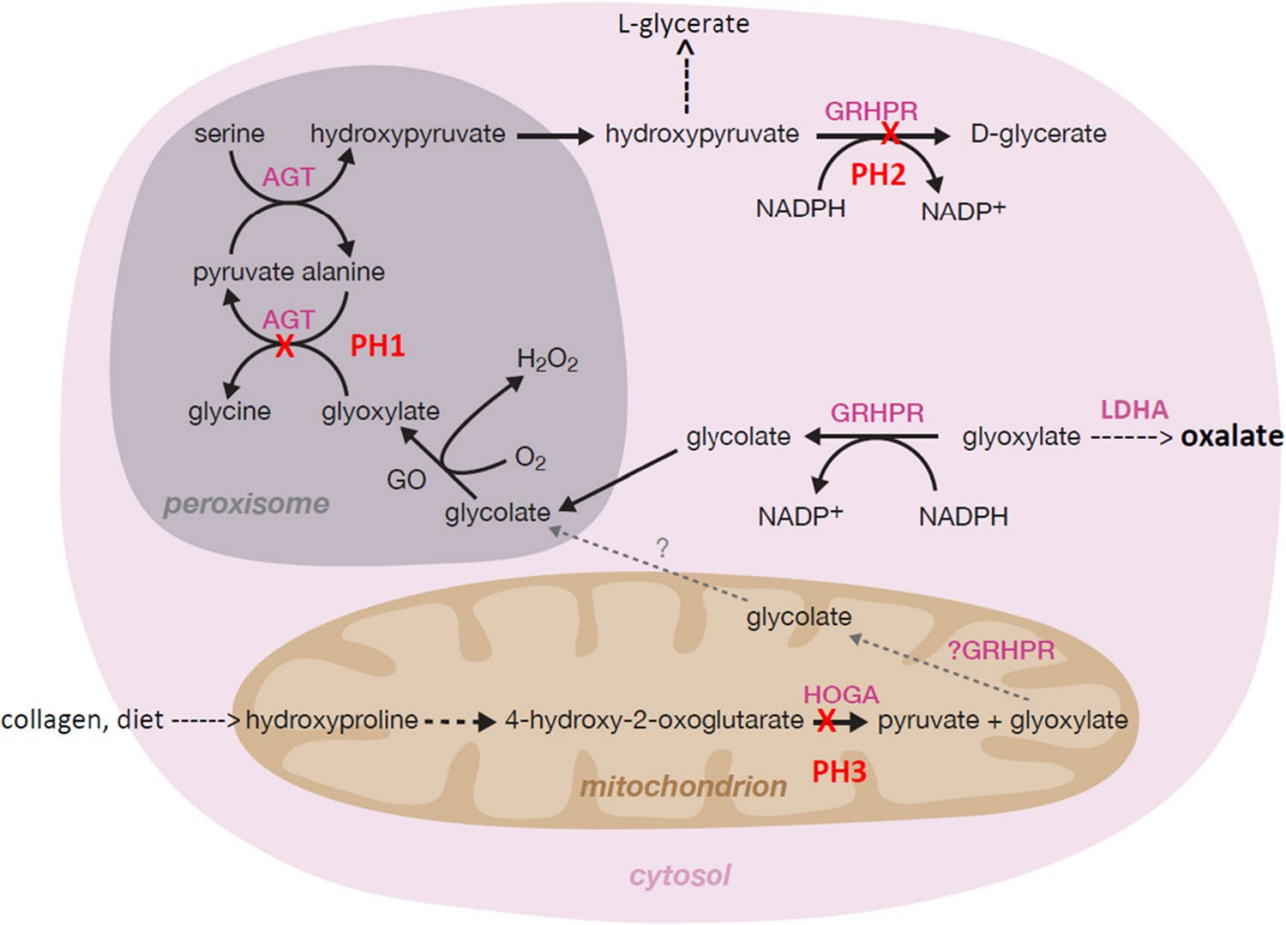
Enzyme deficiencies in glyoxylate metabolism of PH1, PH2, and PH3

Hyperoxaluria refers to excessive urinary excretion of oxalate and is caused by either an increased intestinal absorption of oxalate found in fat malabsorption diseases (secondary hyperoxaluria) or an increased endogenous production of oxalate (primary hyperoxaluria, PH). PH forms a group of autosomal recessive disorders of glyoxylate metabolism in the liver (Fig. 1), which results in an overproduction of oxalate, an end product of metabolism that is almost exclusively excreted by the kidneys. Increased kidney oxalate exposure may cause calcium oxalate crystal formation and subsequently kidney stones or nephrocalcinosis. Oxalate is toxic to the kidney and may induce tubular-interstitial inflammation, eventually causing severe kidney failure particularly in patients with PH type 1 (PH1). Severe kidney failure can occur anytime between the first months and the sixth decade of life, but occurs in 50% of cases before the age of 25 years in PH1 [1, 2]. If the kidney function declines below 30 mL/min/1.73 m^2^, oxalate excretion becomes impaired and will accumulate in the body and stores in various tissues (systemic oxalosis) and may cause life threatening multi-organ disease. Bone, eyes, and myocardium are the main targets for oxalate storage, but oxalate crystals have also been found in nerves, joints, skin, bone marrow, soft tissues, and the liver [3–5]. In the most severe cases of PH, namely, PH1, the overall prognosis has dramatically improved with the recent approval of RNA interfering substrate-reducing therapies enabling an alternative to combined liver-kidney transplantation (CLKT) [6].

There are three types of PH with a well-defined causative metabolic defect and associated mutations. However, the clinical and biochemical phenotype is extremely heterogeneous, with a high discordance within families, where one member may have infantile onset of kidney failure and another carrying the same mutation, preserved kidney function, up to late adulthood [7]. Unfortunately, biochemical assessment of PH has many hurdles, such as high biological variation of oxalate excretion which may exceed 33% [8], and, in patients who present with kidney failure, only plasma oxalate can be assessed, which is an even more unreliable tool due to systemic storage of oxalate and high analytical variation [9].

PH1 has an estimated prevalence from 1:1,000,000 to 3:1,000,000 depending on the population studied [10]. This may be even higher since population analysis by whole genome sequencing indicates an inferred prevalence of 1:121,499 for PH1, 1:196,952 for PH2, and 1:79,499 for PH3 [2].

In this paper, we will review the current knowledge on the relation between genotype on the one hand and biochemical and clinical phenotype on the other hand in PH and discuss the diagnostic and therapeutic implications for certain genotypes.

## PH type 1: biochemical and clinical phenotype

PH1 is caused by a deficiency of the liver-specific, peroxisomal, pyridoxal phosphate-dependent enzyme, alanine:glyoxylate aminotransferase (AGT) which is encoded by the *AGXT* gene [11, 12]. When AGT is deficient, peroxisomal glyoxylate cannot be metabolized into glycine and diffuses to the cytosol where it is subsequently converted into oxalate by LDH or glycolate by glyoxylate reductase/hydroxypyruvate reductase (GRHPR), respectively [13]. This newly formed glycolate can be re-oxidized by the peroxisomal enzyme glycolate oxidase (GO) to glyoxylate and consequently oxalate. Thus, there is overproduction of both oxalate and, in ≈75% of patients, of glycolate [14]. A minority of PH1 patients have normal glycolate excretion, possibly due to increased activity of glycolate oxidase (Fig. 1).

PH1 accounts for 80% of all PH patients and has the most severe clinical phenotype. Over, 70% of patients will develop kidney failure at a certain point. Clinical appearance is, however, extremely heterogeneous and may vary from infantile onset of kidney failure with signs of systemic oxalosis to late kidney failure in a patient with recurrent lithiasis or nephrocalcinosis. In some patients, the diagnosis is only established after recurrence following an isolated kidney transplant. Systemic oxalosis corresponds to a multi-organ disease caused by systemic oxalate storage. In infants, eyes, heart, and bone are particularly vulnerable to oxalate toxicity; in older patients with severe kidney disease due to PH, bone and heart are most often involved [3, 4, 15].

## Most common mutations in PH type 1

AGT occurs in humans as two polymorphic forms known as AGT-major (AGT-Ma) and AGT-minor (AGT-Mi); the latter occurs with an overall population frequency of 0.14 (SNP database: https://www.ncbi.nlm.nih.gov/snp/) but is around 0.46 among PH1 patients [16]. The AGT-Mi protein is characterized by a proline to leucine change at codon 11, and, although this does not lead to disease, it encodes a weak mitochondrial targeting and reduces AGT activity by 30% [17]. Some of the most frequent mutations, such as p.G170R, p.F152I, p.G41R, and p.I244T, occur almost exclusively on the background of the AGT-Mi, and this change contributes to their dysfunctional effect.

Over 200 causative mutations have been identified in *AGXT*, which are associated with a wide variety of effects on AGT. These effects include loss of catalytic activity, peroxisome-to-mitochondrion mistargeting, accelerated enzymatic degradation, and AGT protein aggregation [18, 19]. The most frequent mutation in PH1 is p.G170R that occurs in *cis* with the minor allele and accounts for 28–30% mutant alleles [2, 20–22] (Table 1). This variant is associated with mistargeting of the AGT protein to the mitochondrion instead of the peroxisome [23].

**Table 1:** Overview of most frequent PH1, PH2, and PH3 mutations

Frequent mutations of Primary hyperoxaluria					
PH type	Gene	Mutation	Protein	Pyridoxine sensitive	Predominant region or ethnicity
1	*AGXT*	c.508G > A	p.G170R	+	Caucasian
		c.33dupC	p.K12fs	-	N/A
		c.731 T > C	p.I244T	±	Northern Africa and Canary Islands
2	*GRHPR*	c.103delG	p.D35TfsTer11	N/A	Caucasian
		c.404 + 3_404 + 6del	Missplicing, p.?	N/A	Asian
		c.494G > A	p.G165D	N/A	Asian
3	*HOGA1*	c.700 + 5G > T	Missplicing, p.?	N/A	European
		c.834_834 + 1GG > TT	Missplicing, p.?	N/A	Chinese
		c.944_946delAGG	p.E315del	N/A	Ashkenazi Jewish

Corresponding literature and references can be found in main text. *N/A* not applicable

Two other variants, c.33dupC and p.I244T, account for 12–15% [2, 20] and 6% [20] respectively, although the latter occurs more frequently in populations of Spanish and North African descent with frequencies as high as 84% in unrelated Moroccans [2, 24] and 92% in the Canaries [25]. Other ethnic groups may show a completely different spectrum of mutations: For example, the p.G170R variant is rare in individuals of Pakistani descent in whom p.G350D is more common and is notably absent in the Chinese population in which c.33-dupC is more prominent with little overlap with the Caucasian pattern of mutations [2, 26–28].

## Relation between genotype and phenotype in PH1 and the role of vitamin B6

The large number of individual mutations makes meaningful comparison of genotype– phenotype difficult. The clinically most important group of mutations are the missense mutations that induce mistargeting of the AGT enzyme to the mitochondrion instead of the peroxisome. In some of these mutations, the mistargeting can be (partly) restored by vitamin B6 (pyridoxine–VB6), a cofactor of AGT.

Current recommendations suggest testing all PH1 patients for VB6 responsiveness [29]. Valid data on VB6 response are scarce as all PH1 patients are treated with pyridoxine from the moment of clinical suspicion of PH1, and stopping or weaning is considered not to be ethical in patients with assumed VB6 sensitivity, based on the found mutation. Only one trial has been performed in 12 patients [30]. From this study and other observations, it is expected that approximately about 10–20% of PH1 patients completely normalize their urinary oxalate excretion (Uox) in response to pyridoxine, 30% may experience a partial response, and 50–60% would be refractory [31], at least in Western Europe and the USA where pyridoxine-sensitivity–associated mutations are more prevalent than in other parts of the world. Many attempts have been made to correlate the genotype and the pyridoxine response, either in clinical settings or in cellular models. The available data come from small retrospective analyses or cohort studies and are often based on circumstantial evidence, such as clinical response and a poor understanding of the biological variation of oxalate. Of some mutations, there is quite robust clinical evidence of *in vivo* responsiveness; in others, the VB6 responsiveness has only been established in vitro, while *in vivo* data are inconclusive or negative.

### Mutations with evidence for *in vivo *VB6 responsiveness

#### p.G170R-Mi and p.F152I-Mi

Fargue et al. expressed PH1-associated variants in Chinese hamster ovary (CHO) cells cultured in a medium mimicking plasma VB6 concentration and found that pyridoxal 5′-phosphate (PLP) increased catalytic activity and protein levels and promoted peroxisomal import [32]. For the p.G170R mutation, they demonstrated that increasing the VB6 concentration in the culture medium resulted in a significant increase in the net expression level and catalytic activity, because VB6 rescues this mutant from rapid degradation. The increase in catalytically active peroxisomal AGT explains the reduction of urinary oxalate, seen in p.G170R patients. In the same cellular model, the p.F152I mutation behaved similarly to p.G170R in terms of net expression and intracellular localization, but it had a very low activity in the absence of added PLP; the amount of apoprotein increased when VB6 concentration increased, probably due to a prosthetic group effect of PLP [32]. Cellini et al. found that the p.F152I variant converts into the apo-form during catalysis and postulated that the response to VB6 might be due to an increase of PLP concentration in the liver cytosol, thus shifting the equilibrium of p.F152I-Mi from the apo- to the holo- form counteracting aggregation and mitochondrial mistargeting [33]. In vitro studies have indicated that besides its role as coenzyme, PLP exerts a chaperone function for AGT promoting folding, inducing dimerization, and stabilizing the protein once folded [34–37].

There are data that support *in vivo* VB6 responsiveness of these mutations. In the only formal trial with pyridoxine in 12 PH1 patients, the urinary oxalate decreased by 47% in homozygous p.G170R, somewhat less for p.F152I patients under pyridoxine, 20.9% in heterozygous patients for these mutations, and not significantly in patients with other mutations [30]. In total, more than 30% reduction in Uox was achieved in 50% of patients but without complete normalization of Uox. Data from a Dutch study also indicated a pyridoxine response in those with p.G170R or p.F152I mutations, while a US study showed near to complete normalization of Uox in 6 p.G170R homozygotes and a significant reduction of Uox in 7/8 compound heterozygotes for p.G170R [38, 39]. Indirect evidence for pyridoxine responsiveness based on clinical response comes from registry studies. A large OxalEurope cohort study showed a significantly later onset of kidney failure in patients homozygous for p.G170R or p.F152I compared to PH1 patients with other mutations [1]. A recent registry study showed that overall outcome with respect to patient and kidney graft survival of CLKT was significantly superior to that of isolated kidney transplantation in PH1 patients deemed to be pyridoxine unresponsive based on genotype, but that both transplantation strategies had equal outcomes in homozygous p.G170R and p.F152I patients [40]. A report on three PH patients with kidney failure and homozygous for p.G170R showed that pyridoxine supplementation led to sustained normalization of oxalate excretion after kidney-only transplantation [41].The impact of these findings is extremely important as establishment of full pyridoxine responsiveness may avoid the need for a risky liver transplantation or costly RNAi therapy in such patients. The cohort studies also showed that compound heterozygous patients for p.G170R or p.F152I partly respond to pyridoxine and have an outcome with respect to age at time of kidney failure intermediate to that of pyridoxine unresponsive PH1 patients and homozygous p.G170R/p.F152I patients [1, 2] in line with the lower response of heterozygous patients found in the German trial [30]. Finally, a stable isotope study, using labeled glycolate and glycine showed that compound heterozygous p.G170R/p.F152I or homozygous p.G170R/p.G170R patients had significantly less endogenous oxalate production than VB6 insensitive patients and could, contrary to VB6 insensitive patients and equally to healthy controls, produce glycine out of glycolate, which implies AGT functionality in these patients [42].

### Potential candidate mutations for *in vivo *VB6 responsiveness

VB6 response has been tested in vitro for several other mutations, but most lack clinical confirmation:

#### p.G47R-Mi

This mutation alters subcellular localization, aggregation, and overall stability. Exposure of G47R-mi expressing cells to VB6 increases the expression level and the specific activity in a dose-dependent manner, redirects all the protein to peroxisomes, and increases the ability to detoxify glyoxylate and reduce oxalate production [43].

#### p.I56N-Ma

This mutation reduces the dimer stability in the apo-form and is responsive to VB6. On the contrary, no response was demonstrated in the context of the minor allele. It is possible that the destabilization caused by the concomitant presence of mutations at Ile56 and Pro11 overcomes the rescuing effects of VB6 [44].

#### p.G161R/S/C

Three mutations affecting this residue were reported among PH1 patients: p.G161R on the major allele and p.G161S and p.G161C on the minor allele. These mutations are predicted to interfere with AGT folding promoting protein aggregation. In CHO cells, VB6 is able to reduce protein aggregation, therefore increasing the enzymatic activity [45].

### Point mutations with a so far unclear or negative role of VB6

#### p.I244T-Mi

This leads to an increase in net expression and catalytic activity by VB6 in vitro, and peroxisomal localization is unchanged [25]. Clinical data on VB6-responsiveness for this mutation are scarce and inconsistent [25]. A study from Tenerife showed clinical indication, but no hard evidence of VB6 unresponsiveness; VB6 sensitivity was actually tested in only 1 patient and found to be negative [46].

In a large cohort study of 410 genetically defined PH1 patients, 9 out of 31 homozygous p.I244T were reported to respond to pyridoxine and had significantly more preserved kidney function during follow-up than those who were reported as non-responders [1]. Anecdotally, a sustained response has been described in an individual who was a compound heterozygote for p.I244T/c.847-3C > G (incorrectly reported as 969-3C > G) [47].

#### p.G41R-Mi

This mutation leads to intraperoxisomal aggregation and a partial mistargeting of AGT-Mi. PLP had little or no effect on specific activity, expression level, and subcellular localization of the G41R-mi [48], and the mutant protein has a reduced binding affinity for PLP. A partial but significant response has, however, been reported in a p.G41R-Ma homozygote reducing Uox over 50% [49].

#### p.G82E-Ma

This mutation is associated with preserved immuno-reactive AGT, but with no catalytic activity. It not only reduces the binding affinity for PLP and pyridoxamine 5′-phosphate (PMP), but also causes a catalytic defect related to the transamination step. Therefore, for patients with this mutation, pyridoxine treatment is not expected to be effective [48]. In 2 out of 3 homozygous patients for this mutation of a Dutch cohort, pyridoxine responsiveness was tested and found to be negative, and 2 of them had moderate or severe kidney failure at a relatively early age [39].

## Uncertainties with respect *in vivo* VB6 responsiveness

### Lack of reliable data

There are several reasons for the lack of data on *in vivo* pyridoxine response in patients with these mutations. The first is that they are far more rare than p.G170R which hampers clinical assessment. The two largest PH1 studies [1, 2] did not explore differences in pyridoxine response among various *AGXT* mutations, other than the p.G170R and p.F152I mutations. Therefore, prospective trials to assess the correlation among genotype and pyridoxine response for these mutations are needed. After all, it is possible that other, yet unidentified, pyridoxine responsive genotypes could be identified, as well as dose response studies to define the minimum effective dose of VB6 for each responsive mutation. Detection of pyridoxine response could avoid or delay more invasive procedures (such as liver transplantation) or costly therapies (such as RNAi therapies) and should be particularly useful in low-income countries.

### Pyridoxine not the ideal VB6 derivate?

Pyridoxine, the prescribed formulation of vitamin B6, might be less active in certain mutations than the biological active form of vitamin B6. Vitamin B6 is known to be converted to PLP (the biologically active form) inside the cell. Oppici et al. compared the effect of pyridoxamine (PM) and pyridoxal (PL) with that of pyridoxine in CHO-GO cells expressing the p.G170R-Mi and p.F152I-Mi variants and found that PM or PL increases the glyoxylate detoxification ability of these cells more efficiently than pyridoxine [50]. Moreover, PM and PL are more efficient also for the p.G41R-Ma, p.G161R-Ma, and p.I244T-Mi variants. For p.G41R-Ma and p.G161R-Ma, in the presence of PM or PL, the activity is raised because aggregation is prevented, and the intracellular stability is increased. In the case of p.I244T-Mi, PM and PL increased the protein levels by ∼2.5-fold, and the specific activity increased by 1.6- and 1.4-fold in the presence of PM or PL, respectively. Therefore, based on this in vitro study, clinical studies should also address the role of other B6 vitamers in PH1 patients.

## PH type 2 and type 3

PH type 2 (PH2) is caused by a deficiency of the enzyme glyoxylate reductase/hydroxypyruvate reductase (GR/HPR) [51], which is encoded by *GRHPR.* This enzyme plays a role in the gluconeogenic pathway from serine and in the detoxification of cytosolic glyoxylate. Lack of GR/HPR leads to an accumulation of glyoxylate and hydroxypyruvate, which are both metabolized by lactate dehydrogenase to oxalate and L-glycerate, respectively (Fig. 1). A high L-glycerate and oxalate excretion is the biochemical hallmark of PH2. Contrary to PH1, calcium and citrate excretion are often not decreased. The clinical course is more benign than that of PH1, but eventually, 25% of patients develop kidney failure [52]. No cases of infantile oxalosis have been reported so far. The age of onset of disease is similar to PH1, but progression to kidney failure is delayed [2, 52].

PH type 3 (PH3) is the most recently discovered subtype of PH and results from loss of function of the cytosolic liver enzyme 4-hydroxy-2-oxoglutarate aldolase (HOGA), encoded by *HOGA1* [53]. HOGA deficiency leads to an elevation of urinary 4-hydroxyglutamate, 2,4-dihydroxyglutarate, and 4-hydroxy-2-oxoglutarate and oxalate (Fig. 1). Why urinary oxalate is increased due to this enzyme deficiency is not completely understood [54]. Urolithiasis is the main clinical feature, and the overall clinical course is the most favorable of all PH types. However, a recent OxalEurope cohort study showed that chronic kidney disease may develop in more than 20% of patients [55], albeit later in life. Urinary oxalate excretion is comparable to PH2 and PH1; hypocitraturia and hypocalciuria are mostly absent. The median age at onset is similar to other forms of PH, around 2 to 3 years for first symptoms [52, 55, 56].

PH2 and PH3 account for approximately 10% of cases of PH respectively.

## Mutations in PH2 and PH3

A single nucleotide deletion, c.103delG, accounts for 31–35% [2, 52] of mutant alleles in PH2 mainly restricted to those of Caucasian descent. A 4-base pair deletion, c.404 + 3_404 + 6del (https://varnomen.hgvs.org, previously known as c.403_404 + 2delAAGT), and p.G165D each occur in 14–18% of alleles [2, 52] of patients with predominantly Asian ethnicity. There is no difference in survival of kidney function between homozygotes for the three most common mutations, c.103delG, c.404 + 3_404 + 6del, and p.G165D [52].

In PH3, one common mutation, a single intronic nucleotide change in intron 5, c.700 + 5G > T, leads to missplicing of mRNA and accounts for around 50% of alleles [57, 58]. The mutation profile differs in those of Chinese descent where a splice site mutation, c.834_834 + 1GG > TT, accounted for 50% alleles in one series with no c.700 + 5G > T found in this cohort [59]. A 3-base pair deletion, p.E315del, is also relatively common, with homozygous cases accounting for 19% of individuals in one cohort [56] although it appears to be restricted to those of Ashkenazi Jewish descent.

There are almost no reports of discordance in PH2 or PH3 with the exception of a single PH3 family where two more severely affected siblings did carry a third pathogenic *AGXT* allele, which could also be a chance finding unrelated to their clinical course [58], although this particular incidence does highlight the importance of identifying two causative mutations to reliably make a genetic diagnosis of PH.

## Discordance: the potential role of modifiers

There are a high number of discordant PH1 families [7] and a highly diverse interfamilial expressivity of the PH1 phenotype: Indeed, patients with the same *AGXT* genotype, even within one family, may present with either infantile or late onset CKD stage 5. Therefore, both environmental and genetic factors that are unrelated to the effect of the causative mutation(s), so-called modifiers, have been frequently suspected in PH1. Unfortunately, the phenomenon of discordance has not been studied systematically outside the report of single families [60, 61].

The number of identified cases with definite molecular diagnosis of PH1 in Europe and worldwide would allow linkage studies in discordant families, while the Genome Wide Association Studies (GWAS) (early versus late kidney failure) may identify genomic loci that are associated with kidney survival in PH1 and therefore may be of prognostic or therapeutic relevance.

## Unspecified types of hyperoxaluria

A small group of hyperoxaluric patients lack clear evidence of fat malabsorption and do not have a mutation linked to one of the known PH genes. The intestinal wall could be an alternative target for genetic changes that may induce hyperoxaluria in some of these patients. Recently, a patient with calcium oxalate stones was found to have a rare heterozygous missense mutation (c.1519C > T/p.R507W) in *SLC26A6* that encodes a secretory oxalate transporter in the intestine [62]. The transport of oxalate over the intestinal wall is bidirectional. In mice, it has been shown that ablation of the SLC26A6 transport results in decreased oxalate excretion from the blood to the intestinal lumen, resulting in a net hyperabsorption and subsequently hyperoxaluria and kidney stone formation [63, 64]. Cornière et al. demonstrated that the mutation in their patient had a strong dominant-negative effect in vitro, suggesting that the phenotype of patients heterozygous for this mutation could be more severe than predicted by haploinsufficiency alone [62]. Further studies are needed to confirm these findings.

## Prenatal diagnosis

Prenatal diagnosis may prevent early onset of severe disease in some patients and may even have become more important with the arrival of the new RNAi therapies, as a recent paper has illustrated [65]. Couples undergoing prenatal diagnosis (PND) or preimplantation genetic testing (PGT) should be counselled by a qualified health professional with experience in PH, who can explain the benefits and the risks of both PND and PGT, taking into account outcome and therapeutic options. As with all genetic counselling, discussions should be non-directive, avoiding jargon and incorporate the couple’s cultural beliefs where possible. The decision to have prenatal diagnosis belongs only to the couple [66]. Couples interested in PGT should be offered their first counselling session with an expert in PH and then an expert in PGT to obtain all the information useful to make an informed choice. If the couple wishes to pursue PGT, confirmation of the genetic diagnosis should be offered, either by chorionic villus sampling or newborn DNA testing. PGT should be offered and performed in accordance with international recommendations [67].

Preconception counselling may be offered to couples known to be high risk of PH as well as in the first weeks of a high-risk pregnancy to make an informed decision. Diagnosis is typically made on chorionic villus biopsy at 10–12 weeks gestation.

Fetal-cell-free DNA screening is not currently recommended for autosomal recessive diseases because there are not sufficient data to provide information regarding accuracy and positive and negative predictive value in the general population [68].

Genotype testing of the partner of a heterozygous subject is not recommended unless the couple are blood relatives as the risk of an affected child to an unrelated couple where one is a carrier is low (Table 2).

**Table 2:** Examples to illustrate the recurrence risk in a rare autosomal recessive disorder like PH. A carrier rate 1:150 in the general population was arbitrarily set and is compatible with the estimated prevalence of PH1. The a posteriori risk can be significantly reduced on exclusion of the familial mutation and negative testing of the complete coding sequence of the relevant gene. A priori refers to the chance for having a child with PH with no knowledge before testing of the genetic constitution of the partner, a posteriori to the chance of having a child with PH with the knowledge of genetic constitution after testing of the partner

Carrier frequency	Estimated prevalence	“Low-risk” partner	“High-risk” partner	Risk of affected child	
				A priori	A posteriori*
Rare 1:150	1/90000	Unrelated individual	Affected (biallelic)	1 × 1/150 × 1/2 = 1/300 0.33%	1/6000 ≈0.02%
			Confirmed carrier	1 × 1/2 × 1/150 × 1/2 = 1/600 0.17%	1/1200 ≈ 0.01%
			Second-degree relative of affected patient (e.g., aunt)	1/2 × 1/2 × 1/150 × 1/2 = 1/1200 0.08%	**
		First-degree cousin	Affected (biallelic)	1 × 1/4 × 1/2 = 1/8 12.5%	1/6000 ≈ 0.02%
			Confirmed carrier	1 × 1/2 × 1/8 × 1/2 = 1/32 3.13%	1/1200 ≈ 0.01%
			Second-degree relative of affected patient (e.g., aunt)	1/2 × 1/2 × 1/8 × 1/2 = 1/64 1.56%	**

Asterisk (*) denotes calculation with 95% analytical sensitivity of the assay, assuming negative result for “low-risk” partner. Double asterisks (**) indicate equivalent a posteriori risk to the scenario above (modified from [71])

## Family members at risk and rationale for carrier testing for PH 1–3 in families

The prototypic textbook scenario of a genetic consultation in autosomal recessive PH 1–3 is that of a couple with an affected child wishes to have further children. If both partners are confirmed PH (1–3) carriers, the recurrence risk is 25% (“1 in 4”).

In the real world, we encounter many variations of this scenario (we have listed some in Table 2 for the purpose of risk illustration before and after using an arbitrarily set carrier frequency of 1:150 in the general population compatible with a rare autosomal recessive disease).

Apart from the carrier rate, consanguinity between the partners has profound effects on the risk to offspring. Not surprisingly, the rarity of the disease has no bearing on the a priori risk in consanguineous couples, and as the degree of kinship to the index patient of the family decreases, the a priori risk of recurrence decreases as well. The highest recurrence risk for an affected offspring will be in cases of a so called pseudo-dominant inheritance pattern, as may occur in PH [61] (see examples in Table 2). Pseudo-dominance describes the situation in which the inheritance of a recessive trait mimics a dominant pattern, characterized by a vertical pattern of inheritance in two or more generations of the pedigree. Such is seen when an individual affected with a recessive condition and an unaffected carrier give birth to a child with the same recessive disorder as the affected parent, a situation which particularly occurs in families with multiple consanguineous marriages.

The problem with carrier testing (a predictive test) in autosomal recessive diseases like PH is the lack of practical guidance concerning which scenarios should become an indication for an individual carrier test and what risks are considered too low to be further clarified, but Table 2 can be useful for counselling. Predictive genetic testing in asymptomatic individuals especially children (excluding carrier testing) should only be carried out after consultation with a geneticist and following appropriate counselling for the family.

Genetic counseling in siblings of an affected child is highly encouraged even if asymptomatic, as biochemical testing alone is not always reliable in children and the interfamilial phenotypic variability can be high [69]. The availability of new RNAi therapies make it even more important to detect PH early in siblings of PH patients, as these therapies may prevent kidney injury by PH if applied in a timely way.

## Conclusions

Establishment of the diagnosis PH by genetic testing is extremely important as it has direct impact on the clinical management of the patient as well as implications for family planning in those of childbearing age. Prompt diagnosis is crucial in PH1 patients to facilitate early treatment and prevent kidney failure. Patients who present with kidney disease may be VB6 responsive, but the biochemical assessment of B6 sensitivity can be difficult due to systemic oxalate storage. Genetic testing may have a direct impact also on the choice for combined liver kidney transplantation or kidney alone transplantation. Genetic testing is also mandatory for selecting patients for the new RNAi therapies as efficacy has so far only been established in PH1 patients [6, 70].
